# Psychological Intimate Partner Violence Across Identities: Preliminary Psychometrics of the Multidimensional Measure of Emotional Abuse-Short Form Among Heterosexual, Lesbian, and Bisexual Samples in Türkiye

**DOI:** 10.1007/s10508-026-03422-4

**Published:** 2026-04-28

**Authors:** Ezgi Toplu Demirtaş, Burcu Zurnacı

**Affiliations:** 1 Department of Psychological Counseling and Guidance, MEF University, Istanbul, Turkey; 2 Department of Clinical, Social, and Intercultural Psychology, University of Padova, Via Venezia, 12, 35131 Padua, Italy; 3 Department of Psychological Counseling and Guidance, MEF University, Istanbul, Turkey; 4 Department of Psychology, MEF University, Istanbul, Turkey

**Keywords:** Psychological violence, Intimate partner violence, Sexual and romantic relationships, Sexual minorities, Sexual orientation

## Abstract

Psychological intimate partner violence (IPV) affects individuals across diverse sexual identities and is often reinforced by romantic myths. Despite its substantial mental health consequences, psychological IPV remains under-recognized and insufficiently researched. One barrier is the scarcity of brief, inclusive, and psychometrically sound tools for assessing psychological IPV across varied populations. To address this gap, the present study aimed to validate the short form of the Turkish version of the Multidimensional Measure of Emotional Abuse (MMEA-TR). This 16-item scale evaluates four subdimensions of psychological IPV (Restrictive Engulfment, Denigration, Hostile Withdrawal, and Dominance/Intimidation) while preserving the original multidimensional framework. Data were collected from four independent samples in Türkiye: Bisexual individuals (*n* = 230, *M* = 22.88, *SD* = 4.49), predominantly heterosexual women (*n* = 237, *M* = 24.30, *SD* = 2.51), lesbian and bisexual women (*n* = 178, *M* = 29.06, *SD* = 7.81), and predominantly heterosexual men (*n* = 160, *M* = 24.49, *SD* = 2.54). Confirmatory factor analyses across samples supported the hypothesized four-factor model and demonstrated acceptable fit indices. Subscales showed significant intercorrelations and concurrent validity was supported by theoretically consistent associations with jealousy (Study 1), anxious attachment (Study 2), internalized heterosexism (Study 3), and fragile masculinity (Study 4). Internal consistency reliability ranged from acceptable to high across groups. Overall, findings provide initial evidence for the construct validity, concurrent validity, and reliability of the MMEA-TR short form. This study contributes a brief, inclusive, and psychometrically robust instrument for assessing psychological IPV in both heterosexual and sexual minority populations.

## Introduction

Psychological violence encompasses coercive and aversive behaviors intended to harm or threaten an individual’s emotional well-being or sense of self (Murphy & Hoover, 1999) and is consistently shown to be closely linked with other forms of intimate partner violence (IPV). For example, it may precede physical violence (Murphy & Cascardi, 1999), and recent meta-analytic evidence indicates strong associations between psychological IPV perpetration and both victimization and perpetration of other IPV forms (Palmer et al., 2024). Psychological violence is widespread and has been consistently reported as the most prevalent form of IPV across diverse populations. Among heterosexual individuals, rates of psychological IPV perpetration have reached as high as 60% (Graña et al., 2017). Similarly, among lesbian, gay, bisexual, and transgender (LGBT) individuals, perpetration of psychological violence is the most frequently reported form of violence (Kimmes et al., 2017; Trujillo‑Guablocho et al., 2025).

Despite its high prevalence, psychological violence may be normalized within romantic relationships through the endorsement of romantic ideals. Ideals such as “one and only,” “love at first sight,” or the idea that “love overcomes all obstacles” can make it difficult to recognize abuse in relationships (Sprecher & Metts, 1989). For example, some women may idealize their abusive partners and interpret controlling behavior as signs of love (Power et al., 2006; Wood, 2001). Moreover, psychological IPV often receives less attention and is underestimated compared to physical or sexual IPV by people (Sikström et al., 2021; Stern et al., 2019). For instance, among college students, the invisibility of harm leads to psychological IPV being viewed as less severe than physical or sexual forms (Mclennan et al., 2025).

Psychological IPV experiences can increase traumatic stress, thereby contributing to adverse mental health outcomes, including depression, anxiety, and posttraumatic stress disorder. Indeed, Dokkedahl et al. (2022) found that psychological IPV involving coercive control showed the strongest association with posttraumatic stress disorder among all IPV forms. Similarly, White et al. (2023) demonstrated that the impact of psychological IPV on psychological distress can be comparable in magnitude to that of physical and sexual IPV. In addition, a comparison of lesbian, gay, and bisexual (LGB) and heterosexual IPV survivors revealed that, when exposed to forms of psychological violence such as verbal threats, LGB survivors had significantly higher rates of being diagnosed with depression and anxiety compared to their heterosexual counterparts (Miller & Irvin, 2016).

Briefly, although psychological IPV is common, linked to other forms of IPV, often normalized under romantic ideals, perceived as not abusive, and related to substantial mental health harm, it remains under-recognized, and studies on psychological IPV are still insufficient relative to its public health importance, especially in Türkiye across both heterosexual and LGB samples and internationally within diverse samples. This may be partly attributable to the lack of concise, psychometrically robust, and inclusive instruments that can assess both perpetration and victimization in heterosexual and LGB samples. This gap is also reflected in recent research, which shows that non-Western contexts and sexual minority individuals remain underrepresented in the psychological IPV literature, underscoring the need for culturally and demographically inclusive assessment tools (Hilton et al., 2024; Wesenberg et al., 2025). One reason this need remains challenging to address is the complex and multidimensional nature of psychological IPV, which necessitates lengthy instruments capable of capturing several distinct yet interrelated forms of psychological abuse. Consistent with this multidimensionality, prior research has identified several core domains of psychological IPV, such as Restrictive Engulfment, Denigration, Hostile Withdrawal, and Dominance/Intimidation, that together capture the breadth of psychological abusive behaviors (e.g., Murphy & Hoover, 1999; Tolman, 1989). Therefore, instruments designed to address the domains of psychological IPV are typically lengthy. For example, widely used multidimensional psychological IPV instruments such as the Multidimensional Measure of Emotional Abuse (MMEA) (28 items; Murphy & Hoover, 1999) and the Psychological Maltreatment of Women Inventory 58 items; Tolman, 1989) demonstrate strong psychometric performance, including stable factor structures and high internal consistency coefficients (often in the .80–.90 range). However, these instruments require substantial administrative time due to their length. In large-scale studies, such length may increase participant burden, reduce completion rates, and decrease data quality. Therefore, a brief yet multidimensional tool is particularly advantageous when working with diverse samples or time-limited survey designs. The MMEA short form (Maldonado et al., 2022) aligns with this aim, but its psychometric properties have not been evaluated in Turkish or among diverse samples worldwide. Therefore, this study addresses that gap by assessing the construct and criterion-related validity of the short form of the MMEA in Türkiye across multiple independent samples: Heterosexual and lesbian and bisexual individuals, as well as men and women. To our knowledge, this is the first study to do so, offering a novel and much-needed contribution to the literature.

### Multidimensional Measure of Emotional Abuse

The MMEA stands out from other measures of psychological abuse (Psychological Maltreatment of Women Inventory, Tolman, 1989; Conflict Tactics Scale-2, Straus et al., 1996, etc.) due to several key features that enhance its utility in capturing the complexity of psychological violence in partner relationships. To elaborate, the MMEA, specifically designed to assess psychological violence both among “college and community” and “dating and married” samples and developed within both feminist and behavioral theoretical frameworks, incorporates a multidimensional structure that enables the evaluation of various forms of psychological violence in both victimization and perpetration for men and women, thereby distinguishing it from other measures. Moreover, the use of the gender-neutral term partner ensures that the scale is applicable across diverse sexual orientations. Additionally, the strong psychometric properties of the original scale have been well documented, with internal consistency coefficients ranging from .83 to .92 and a four-factor structure accounting for approximately 55% of the total variance (Murphy & Hoover, 1999). The original validation also provided evidence for convergent and discriminant validity, as all subdimensions correlated significantly with physical aggression (*r*s = .29–.74). Cross-measure comparisons have further shown that the MMEA performs more effectively than other psychological IPV instruments, demonstrating higher and more stable internal consistency (*α*s = .88–.91; Ro & Lawrence, 2007; *α*s = .80–.94; Shorey et al., 2012) relative to the Conflict Tactics Scale-2-Psychological Aggression Subscale, whose reliability coefficients are substantially lower and more variable (*α*s = .54–.77; Ro & Lawrence, 2007; *α*s = .29–.64 in frequency scoring; Shorey et al., 2012). The validity of the scale has also been supported across different cultural contexts (Bonechi & Tani, 2011), and the initial psychometric evaluation of the Turkish adaptation (long form) yielded promising results, with the perpetration form demonstrating acceptable reliability across subscales (*α*s = .68–.83) and a four-factor structure supported by adequate confirmatory factor analysis (CFA) fit indices (*χ*^*2*^/d*f* = 2.16, RMSEA = .07, SRMR = .07; Toplu-Demirtaş et al., 2018). More recently, the scale has also been employed to assess psychological violence in LGB populations (e.g., Ummak et al., 2022; Zurnacı & Toplu-Demirtaş, 2025).

In light of these psychometric and conceptual advantages, the MMEA provides a self-report framework for assessing psychological violence in romantic relationships. The original pool of 54 items was developed from multiple theoretical sources and existing emotional abuse measures, and several items were revised or newly created to ensure their relevance to dating relationships. Items that were not applicable to these relationships, such as those assessing economic abuse, male-specific dominance, or minimization of physical aggression, were removed (Murphy & Hoover, 1999). Through subsequent psychometric screening, this initial pool was reduced to a final set of 28 items that best represented the four theorized forms of psychological abuse (Murphy & Cascardi, 1999). These 28 items were selected based on empirical criteria, including having adequate response frequency (i.e., being endorsed by at least 10% of participants), meaningful contribution to subscale internal consistency, strong primary loadings on the intended factor, and clear differentiation from other factors. The first subscale, Restrictive Engulfment, involves attempts to limit and control a partner’s activities and social interactions through jealousy and possessiveness, to foster dependency (e.g., “Tried to stop the other person from seeing certain friends or family members”). The second subscale, Denigration, encompasses humiliating and demeaning behaviors, along with verbal violence, intended to diminish the partner’s self-esteem and sense of self-worth (e.g., “Called the other person a loser, failure, or similar term”). The third subscale, Hostile Withdrawal, refers to deliberately avoiding the partner during conflict and withholding emotional engagement to evoke anxiety or insecurity about the relationship (e.g., “Became so angry that they were unable or unwilling to talk”). The fourth subscale, Dominance/Intimidation, includes damaging property and using verbal violence to create fear and force the partner into submission (e.g., “Threw, smashed, hit, or kicked something in front of the other person”).

### Short Form of the Multidimensional Measure of Emotional Abuse

Recently, a short form of the MMEA (Maldonado et al., 2022) was created to reduce assessment burden while retaining the conceptual structure and psychometric strengths of the original 56-item version (28 items for victimization and 28 items for perpetration). The short form of the MMEA consists of 32 items (16 for victimization and 16 for perpetration) and retains the original four subscales (i.e., Restrictive Engulfment, Denigration, Hostile Withdrawal, Dominance/Intimidation), each comprising four items. CFA results supported the four-factor structure in both perpetration and victimization across clinical and college samples, with acceptable to good model fit. The short form of the MMEA demonstrated acceptable to high internal consistency across all subscales and total scores, with Cronbach’s alpha ranging from .78 to .92 in the clinical sample and from .67 to .91 in the undergraduate sample. In addition, the short form of the MMEA showed expected associations with related constructs such as physical assault and relationship satisfaction, further evidencing its criterion-related validity.

### Links to the Multidimensional Measure of Emotional Abuse

Jealousy is typically defined as an affective, cognitive, and behavioral response to perceived threats to a valued relationship (Pfeiffer & Wong, 1989). Among bisexual individuals, researching jealousy is particularly important because they are uniquely exposed to binegative stereotypes that position them as inherently unfaithful or unreliable, which can heighten relational insecurity and mistrust (e.g., McCole & Anderson, 2025). Jealousy is also one of the most robust correlates of psychological IPV perpetration, consistently linked to behaviors such as controlling, isolating, belittling, emotional withdrawal, and coercive intimidation (Brem, 2025; Keilholtz et al., 2023; Sarno et al., 2024; Velotti et al., 2018, 2020; Zurnacı & Toplu-Demirtaş, 2025). In sociocultural contexts shaped by strong honor norms, including Türkiye, jealousy is often socially sanctioned and even interpreted as an expression of love, potentially legitimizing harmful behavioral expressions of jealousy (Karakoçoğlu & Hasdağ, 2025; Toplu-Demirtaş, 2017). Together, these dynamics suggest that jealousy may operate as an especially potent antecedent of psychological IPV perpetration among bisexual individuals.

Anxious attachment, defined by heightened fears of rejection, preoccupation with a partner’s availability, and a persistent need fhor reassurance (Fraley & Shaver, 2000; Fraley et al., 2000), is a well-established psychological process associated with elevated risk for psychological IPV perpetration (Aracı-İyiaydın et al., 2022; Arseneault et al., 2024; Hazan & Shaver, 1987; Cheche-Hoover & Jackson, 2019; Leclerc et al., 2022; Miyagawa & Kanemasa, 2023; Toplu-Demirtaş et al., 2019; Velotti et al., 2018, 2020). Across LGB and heterosexual populations, empirical work consistently identifies anxious attachment as a significant relational vulnerability predicting the use of psychological aggression (e.g., Toplu-Demirtaş et al., 2018; Trombetta et al., 2024). This association is particularly evident among predominantly heterosexual women, for whom higher attachment anxiety reliably corresponds to greater engagement in psychological IPV perpetration (e.g., Murphy & Hoover, 1999; Toplu-Demirtaş et al., 2018).

Internalized heterosexism, the internalization of negative societal attitudes toward same-gender attractions (Meyer & Dean, 1998), is another key mechanism associated with psychological IPV perpetration, particularly among sexual minority women. Such internalized stigma can erode lesbian and bisexual women’s sense of connection to queer communities, diminish access to social support, and intensify relational isolation, thereby increasing vulnerability to psychological aggression (Balsam, 2001; Balsam & Szymanski, 2005). These internalized beliefs often heighten partner dependence and reduce self-esteem, which may normalize or reinforce abusive dynamics (Badenes-Ribera et al., 2019; Bermea et al., 2018; Lewis et al., 2014; Ummak et al., 2022). Given that lesbian and bisexual women face unique intersections of minority stress and gendered relational expectations distinct from those experienced by gay men, internalized heterosexism represents a particularly important factor to consider when examining susceptibility to psychological IPV perpetration (Lewis et al., 2014; Matte & Lafontaine, 2011).

Fragile masculinity, characterized by heightened sensitivity to perceived threats to one’s manhood (Vandello & Bosson, 2013), is an additional relational process associated with psychological IPV perpetration, particularly among men. When masculinity feels tenuous, men may resort to psychologically aggressive behaviors to reassert power or control (Reidy et al., 2014; Türkoğlu, 2013; Vandello & Bosson, 2013). Empirical evidence shows that men who strongly internalize traditional masculine norms are more likely to justify violence, including psychological aggression, as an appropriate response to masculinity threats (Moore et al., 2010; O’Donnell et al., 2024). In honor culture contexts, these expectations are intensified, as even subtle masculinity threats are seen as warranting defensive or aggressive action (Öztemür & Toplu-Demirtaş, 2024; Uskul & Cross, 2019). Consistent with Gender Role Stress Theory, such reactivity reflects insecurity about meeting culturally prescribed masculine standards (Eisler & Blalock, 1991; O’Neil, 1981). Given that boys and men are socialized across family, peer, institutional, and sociocultural contexts to equate masculinity with dominance and legitimized aggression (Connell & Messerschmidt, 2005; O’Donnell et al., 2024), fragile masculinity may represent a particularly salient contributor to men’s psychological IPV perpetration.

### Current Study

Although the MMEA has been adapted into Turkish in its original form and successfully used with both heterosexual (e.g., Toplu Demirtaş et al., 2018; Toplu-Demirtaş & Hatipoğlu-Sümer, 2023) and LGB samples (e.g., Ummak et al., 2022; Zurnacı & Toplu-Demirtaş, 2025), no validated short‑form version currently exists for use in Türkiye. The MMEA-TR short form is crucial because it preserves the multidimensional structure of the scale, while reducing respondent burden, allowing for its inclusion in large-scale surveys and time-limited research designs. The brevity and inclusive design of the short form make it particularly suited for diverse relational contexts, including both dating and married couples, as well as for research with sexual minority populations, without introducing heteronormative bias.

Therefore, in this study, we aim to evaluate the psychometric properties of the short form of the MMEA-TR (Maldonado et al., 2022) among heterosexual, lesbian, and bisexual samples in Türkiye. To this aim, we examined its factorial validity using CFA, assessed its internal consistency, and tested criterion‑related validity through theoretically relevant correlates such as jealousy for the bisexual sample (Study 1), anxious attachment for the predominantly heterosexual women (Study 2), internalized heterosexism for the lesbian and bisexual women sample (Study 3), and fragile masculinity for the predominantly heterosexual men sample (Study 4) across four studies.

### Study 1

#### Purpose of the Study and Hypotheses

In Study 1, our purpose was to establish initial evidence for the construct validity, concurrent validity, and internal consistency reliability of the MMEA-TR short form among a sample of bisexual individuals. We formulated hypotheses for concurrent validity.

##### ***Hypothesis 1***

All potential sub-factors of the MMEA-TR short form will be correlated with one another among a sample of bisexual individuals.

##### ***Hypothesis 2***

Jealousy will be correlated with all the potential sub-factors of the MMEA-TR short form among a sample of bisexual individuals.

## Method

### Participants

The sample included 230 bisexual individuals from Türkiye, with ages ranging from 18 to 43 years (*M* = 22.88, *SD* = 4.49). Most identified as cisgender women (65.2%), while smaller percentages identified as cisgender men (4.8%), nonbinary (7.8%), gender-neutral/agender (6.1%), transgender men (2.6%), transgender women (1.3%), genderqueer (2.6%), genderfluid (7.0%), two-spirit (1.7%), pangender (0.4%), or other gender identities (0.4%). Almost all participants (98.7%) identified as bisexual, with a small number (1.2%) identifying with orientations under the bisexual umbrella, such as pansexual and demisexual. Most were involved in romantic or sexual relationships (60.9%), followed by long-distance relationships (20.4%). Relationship durations varied from 1 to 144 months (*M* = 16.69, *SD* = 21.68). The majority reported being in monogamous (88.3%) and stable (75.2%) relationships with non-same-sex partners (69.6%).

### Measures

**MMEA-TR Short Form.** To gauge psychological IPV perpetration, we employed the short form of the MMEA (16 items; Maldonado et al., 2022), adapted from the original 28-item MMEA (Murphy & Hoover, 1999). The short form of the MMEA comprises four subscales, each with four items, designed to capture distinct forms of psychological aggression. Participants responded on a 7-point frequency scale (0 = never happened to 6 = more than 20 times), with an additional option for behaviors that did not occur in the past six months. Higher scores reflect more frequent perpetration of psychological IPV. In the Turkish adaptation of the long version, Toplu-Demirtaş et al. (2018) reported Cronbach’s alphas ranging from .68 to .83 across the subscales.

**Multidimensional Jealousy Scale.** To assess jealousy, we utilized the cognitive jealousy subscale from the multidimensional jealousy scale, initially developed by Pfeiffer and Wong (1989). The subscale consists of 8 items and evaluates jealousy-related thoughts, with responses rated on a 7-point Likert scale ranging from 1 (strongly disagree) to 7 (strongly agree). Higher scores reflect greater levels of cognitive jealousy. In the present study, to ensure inclusivity for bisexual participants, we removed references to the “opposite sex” from the items. The Turkish adaptation of the multidimensional jealousy scale was conducted by Karakurt (2001), who reported a Cronbach’s alpha value of .84 for cognitive jealousy.

### Procedure

We collected data from October to December 2023, following ethical approval from the MEF University Human Research Ethics Committee. Using convenience sampling, we distributed the online survey via Google Forms and recruited participants by posting a recruitment poster with the survey link on several popular lesbian, gay, bisexual, transgender, and queer (LGBTQ+) social media platforms on Instagram and Twitter (e.g., queer community hubs and support/advocacy accounts). Participation was completely voluntary, and we did not provide any monetary or material incentives. After giving informed consent, participants completed the survey in about 15 min. Upon completion, we provided a debriefing form that outlined the aims and hypotheses of the study, explained our procedures for ensuring data confidentiality and erasure, and listed free counseling services available to LGBTQ+ individuals. To protect anonymity, we asked participants to create a pseudonym by combining the first two letters of their first name with the first two letters of their last name, along with their preferred color. We stored the data securely, limited access to the study team, and used it only for this research.

### Data Analysis

To examine the four-factor structure of the MMEA-TR short form, we conducted a CFA using Jamovi (Version 2.3) with data from an independent sample of bisexual individuals. Consistent with Brown’s (2006) recommendations, we assessed model fit using a combination of absolute fit indices (*χ*^2^, SRMR), a parsimony-adjusted fit index (RMSEA), and comparative fit indices (CFI, TLI). Given the known sensitivity of the chi-square statistic to sample size, which often results in the rejection of the null hypothesis, we also calculated the chi-square divided by degrees of freedom (*χ*^2^/df) as a more stable indicator of fit.

We applied the following criteria to determine model adequacy: for RMSEA, we followed Browne and Cudeck’s (1993) guidelines, interpreting values below .05 as indicating a close fit, values between .05 and .10 as reflecting a mediocre fit, and values above .10 as suggesting a poor fit. For the *χ*^2^/df ratio, we adhered to Kline’s (1998) recommendation of values below 3. Regarding SRMR, CFI, and TLI, we used Hu and Bentler’s (1999) cutoffs: SRMR values below .08, and CFI and TLI values above .95, were considered indicative of good model fit. While fit indices offer important information for model evaluation, they are not the only aspect to consider. As emphasized by Brown (2006), model evaluation should also consider the clarity and theoretical coherence of factor structures, as well as the size and statistical significance of parameter estimates when fit indices fall within acceptable ranges.

To demonstrate concurrent validity, we calculated the correlation coefficients between variables. Additionally, we reported Cronbach’s alpha values to assess internal consistency. All correlation and reliability analyses were performed using Jamovi (Version 2.3).

## Results

### Confirmatory Factor Analysis

We obtained a mediocre yet acceptable fit: *χ*^*2*^(98, *N* = 230) = 274.00, *p* < .001, *χ*^*2*^*/df* = 2.79, CFI = .854, TLI = .821, RMSEA = .088 (90% CI = .076–.101), and SRMR = .080. The CFI and TLI values fell below the recommended thresholds (i.e., ≥ .90). The modification index check revealed no substantial model misfit or poorly performing items. As seen in Table 1, standardized factor loadings (*β*) ranged from .543 to .698 for Restrictive Engulfment, .503 to .824 for Denigration, .480 to .909 for Hostile Withdrawal, and .176 to .645 for Dominance/Intimidation at *p* < .001. All factor loadings were statistically significant, indicating that each item effectively represented its intended latent construct. Although Item 2 (“Threatened to hit the other person”) under Dominance/Intimidation had a loading below .30, it was retained because it contributed significantly to the factor, and its removal did not improve the model fit.

**Table 1:** Factor loadings of confirmatory factor analysis (Study 1; *N* = 230)

Factors	Indicator	Estimate	SE	Lower	Upper	Std Estimate
Restrictive engulfment	RE_1	.615	.069	.480	.751	.626
	RE_2	.871	.087	.699	1.044	.698
	RE_3	.946	.121	.707	1.185	.557
	RE_4	.569	.076	.419	.719	.543
Denigration	DEN_1	.702	.093	.520	.885	.507
	DEN_2	.315	.042	.233	.398	.503
	DEN_3	.512	.037	.438	.586	.824
	DEN_4	.375	.033	.309	.442	.703
Hostile withdrawal	HW_1	1.477	.097	1.285	1.669	.844
	HW_2	1.554	.092	1.372	1.735	.909
	HW_3	.535	.073	.392	.679	.480
	HW_4	1.226	.105	1.019	1.433	.699
Dominance/Intimidation	DOM_1	.556	.081	.397	.716	.468
	DOM_2	.071	.026	.018	.123	.176
	DOM_3	.379	.055	.271	.487	.493
	DOM_4	.596	.06	.475	.717	.645

Lower and upper refer to unstandardized estimates that are within the 95% confidence intervals

### Correlation Analysis

As shown in Table 2, all sub-factors of the MMEA-TR short form were significantly and positively correlated with one another. Additionally, jealousy demonstrated significant and positive correlations with each of the sub-factors of the MMEA-TR short form.

**Table 2:** Correlation matrix (Study 1; *N* = 230)

Variables	1	2	3	4	5	6
1. Restrictive engulfment	–					
2. Denigration	.31***	–				
3. Hostile withdrawal	.43***	.29***	–			
4. Dominance/Intimidation	.44***	.58***	.48***	–		
5. Psychological violence	.74***	.62***	.83***	.75***	–	
6. Anxious attachment	.31***	.18**	.18**	.18**	.28***	–
Cronbach alpha	.70	.73	.83	.63	.83	.87

Psychological violence was computed as the sum of the four subscales (Restrictive Engulfment, Denigration, Hostile Withdrawal, and Dominance/Intimidation) to represent overall psychological IPV perpetration

^*^ *p* < .05, ** *p* < .01, *** *p* < .001

### Reliability Analysis

The Cronbach’s alpha coefficients for the MMEA-TR short form subscales were .70 for Restrictive Engulfment, .73 for Denigration, .83 for Hostile Withdrawal, and .63 for Dominance/Intimidation.

## Discussion

The findings from Study 1 provide initial support for the construct and concurrent validity of the MMEA-TR short form among bisexual participants. Despite one item in the Dominance/Intimidation subscale loading slightly below the conventional threshold, its theoretical relevance and significant loading justified its retention, and the overall factor structure remained consistent with the original measure. Reliability estimates were generally acceptable, with only minor variation in the Dominance/Intimidation subscale. Furthermore, the positive associations observed between jealousy and all psychological IPV perpetration align with theoretical expectations, underscoring the role of jealousy in psychological IPV perpetration within bisexual intimate relationships.

### Study 2

#### Purpose of the Study and Hypotheses

In Study 2, our purpose was to establish initial evidence for the construct validity, concurrent validity, and internal consistency reliability of the MMEA-TR short form among a sample of predominantly heterosexual women. We formulated hypotheses for concurrent validity.

##### ***Hypothesis 3***

All potential sub-factors of the MMEA-TR short form will be correlated with one another in a sample of heterosexual individuals.

##### ***Hypothesis 4***

Anxious attachment will be correlated with all the potential sub-factors of the MMEA-TR short form among a sample of heterosexual individuals.

## Method

### Participants

The sample consisted of 237 participants, who completed the survey online in January 2023, via a link circulated on social media platforms. Participants ranged in age from 18 to 38 years (*M* = 24.30, *SD* = 2.51). In terms of gender identity, 72.6% identified as women (*n* = 172), 21.5% as men (*n* = 51), and 5.9% (*n* = 14) selected nonbinary, genderfluid, or agender identities. The majority of participants (89.9%, *n* = 213) reported that their gender identity aligned with the sex assigned to them at birth, while 2.5% stated it did not. Additionally, 18 participants (7.6%) selected the response “My experience of gender is more complex than either of these options.” Regarding sexual orientation, most participants identified as heterosexual (67.5%, *n* = 160), followed by bisexual (24.9%, *n* = 59), gay (3.8%, *n* = 9), lesbian (3.4%, n = 8), and one participant identified as asexual/aromantic (0.4%). The majority reported being in a romantic relationship, with 75.5% indicating “We are dating,” 8.9% stating “We live together but are not married,” and 5.1% reporting “We are engaged.” Among those currently in a romantic relationship, relationship duration ranged widely, with a mean of 27.79 months (*SD* = 53.97). Most participants (95.8%) reported being in monogamous relationships, while a smaller proportion (3.4%) indicated they were generally non-monogamous.

### Measures

**MMEA-TR Short Form.** Psychological IPV perpetration was assessed using the short form of the MMEA-TR. For detailed information regarding the measure, please refer to Study 1.

**The Experiences in Close Relationships Inventory-II.** Participants’ anxious attachment was measured using the 18-item Anxious Attachment subscale (e.g., “I often worry that my partner will not want to stay with me”) of the Experiences in Close Relationships Inventory-II, developed by Fraley et al. (2000) and adapted into Turkish by Selçuk et al. (2005). Participants rated the extent to which each item reflected their feelings and thoughts in romantic relationships on a 7-point Likert scale ranging from 1 (*strongly disagree*) to 7 (*strongly agree*). Higher scores reflect a greater tendency toward anxious attachment. The internal consistency coefficient for the subscale was .86, and test–retest reliability analyses yielded a coefficient of .82 in the Turkish version.

### Procedure

We followed a procedure similar to that of Study 1, with three key differences: (1) ethical approval was obtained from the Bahçeşehir University Human Research Ethics Committee, (2) recruitment did not involve the use of LGBTQ+ social media platforms, and (3) no free counseling services were offered to participants in this study.

At the outset of the study, approval was obtained from the Scientific Research and Publication Ethics Committee of Bahçeşehir University. Data were collected during the fall semester of 2022. The survey was created using Google Forms, and participants accessed it via the survey link. The survey link was distributed through social media platforms such as Facebook, Instagram, Twitter, and WhatsApp, using convenience sampling. Participation was voluntary, and no compensation was provided.

Eligible participants were aged 18–30, identified their sexual orientation as heterosexual, lesbian, gay, or bisexual, and reported being currently or previously in a dating relationship. Completing the survey took approximately 15 min. Participants first completed the Informed Consent Form, followed by the Demographic Information Form, the Experiences in Close Relationships Inventory-II, and the MMEA-SF in Turkish.

After finishing the survey, participants were provided with a debriefing form explaining the purpose of the study and the researcher’s contact information for further questions. Only fully completed responses were collected, ensuring no missing data. To maintain confidentiality and anonymity, all survey responses were stored in a secure Google Drive folder with restricted access to the research team and will be retained for five years in accordance with institutional policy.

### Data Analysis

The statistical procedures and tools used in Study 2 were identical to those employed in Study 1. For details, please refer to the data analysis procedure described in Study 1.

## Results

### Confirmatory Factor Analysis

The model yielded an overall moderate yet adequate fit: *χ*^*2*^(98, *N* = 237) = 286.00, *p* < .001, *χ*^*2*^*/df* = 2.91, CFI = .868, TLI = .838, RMSEA = .090 (90% CI = .078-.102), SRMR = .072. Although CFI and TLI values fell below conventional thresholds (i.e., ≥ .90), inspection of modification indices did not reveal substantial areas of misfit or underperforming items. As detailed in Table 3, standardized factor loadings were statistically significant (*p* < .001), ranging from .312 to .653 for Restrictive Engulfment, .378–.888 for Denigration, .462–.840 for Hostile Withdrawal, and .617–.818 for Dominance/Intimidation, supporting the adequacy of the measurement model in capturing the hypothesized latent constructs.

**Table 3:** Factor loadings of confirmatory factor analysis (Study 2; N = 237)

Factors	Indicator	Estimate	SE	Lower	Upper	Std Estimate
Restrictive engulfment	RE_1	.77	.08	.60	.94	.65
	RE_2	.86	.09	.68	1.05	.64
	RE_3	1.09	.12	.83	1.35	.61
	RE_4	.28	.06	.14	.41	.31
Denigration	DEN_1	.92	.09	.73	1.10	.60
	DEN_2	.69	.04	.61	.78	.88
	DEN_3	.77	.05	.67	.86	.84
	DEN_4	.29	.05	.19	.39	.37
Hostile withdrawal	HW_1	1.39	.11	1.17	1.61	.73
	HW_2	1.57	.10	1.35	1.78	.84
	HW_3	.65	.09	.46	.83	.46
	HW_4	1.44	.12	1.20	1.67	.72
Dominance/Intimidation	DOM_1	.88	.07	.73	1.03	.71
	DOM_2	.53	.04	.44	.61	.73
	DOM_3	.71	.05	.61	.81	.81
	DOM_4	.55	.05	.44	.66	.61

Lower and upper refer to unstandardized estimates that are within the 95% confidence intervals

### Correlation Analysis

As presented in Table 4, all subscales of the MMEA-TR short form exhibited significant and positive intercorrelations. Furthermore, anxious attachment was significantly and positively correlated with all subscales of the MMEA-TR short form, with coefficients ranging from *r* = .18, *p* < .01 (Denigration) to *r* = .37, *p* < .01 (Restrictive Engulfment).

**Table 4:** Correlation matrix (Study 2; *N* = 237)

Variables	1	2	3	4	5	6
1. Restrictive engulfment	–					
2. Denigration	.36**	–				
3. Hostile withdrawal	.43**	.42**	–			
4. Dominance/Intimidation	.34**	.60**	.46**	–		
5. Psychological violence	.70**	.73**	.84**	.77**	–	
6. Anxious attachment	.37**	.18**	.25**	.20**	.33**	–
Cronbach alpha	.65	.79	.79	.81	.86	.93

Psychological violence was computed as the sum of the four subscales (Restrictive Engulfment, Denigration, Hostile Withdrawal, and Dominance/Intimidation) to represent overall psychological IPV perpetration

^*^ *p* < .05, ** *p* < .01, *** *p* < .001

### Reliability Analysis

The Cronbach’s alpha coefficients for the MMEA-TR short form subscales were .650 for Restrictive Engulfment, .790 for Denigration, .790 for Hostile Withdrawal, and .810 for Dominance/Intimidation.

## Discussion

Study 2 further supported the construct and concurrent validity of the MMEA-TR short form in a sample of predominantly heterosexual women. The four-factor structure replicated the original scale, and although one subscale demonstrated slightly lower internal consistency, reliability estimates were generally within acceptable ranges. The significant correlations among the psychological IPV subdimensions indicate that they represent interrelated but distinct forms. Importantly, the associations between anxious attachment and all dimensions of psychological IPV perpetration suggest that attachment insecurity may heighten vulnerability to engaging in psychologically violent behaviors within intimate partnerships.

### Study 3

#### Purpose of the Study and Hypotheses

In Study 3, our purpose was to establish initial evidence for the construct validity and internal consistency reliability of the MMEA-TR short form among a sample of lesbian and bisexual women. We formulated hypotheses for concurrent validity.

##### ***Hypothesis 5***

All potential sub-factors of the MMEA-TR short form will be correlated with one another in a sample of lesbian and bisexual women.

##### ***Hypothesis 6***

Internalized heterosexism will be correlated with all the potential sub-factors of the MMEA-TR short form among a sample of lesbian and bisexual women.

## Method

### Participants

The sample comprised 178 participants, with a mean age of 29.06 years (*SD* = 7.81). In terms of gender identity, the majority identified as cisgender (93.3%), followed by transgender (5.1%) and participants with complex gender identities (1.7%). Regarding sexual orientation, 57.9% of participants identified as bisexual, while 42.1% identified as lesbian. A majority (69.1%) reported being in a current romantic relationship, whereas 30.9% indicated that their most recent relationship had ended. Reported relationship types included dating (26.4%), cohabitation (20.2%), long-distance (14.6%), marriage (6.2%), and engagement (1.1%), with 1.5% selecting “other.” For perceived relationship seriousness, 60.5% described their relationship as stable or serious, 1.3% as casual, 9.2% expressed uncertainty, and 6.6% selected “other”; additionally, 22.4% reported not currently being in a romantic relationship. For those in ongoing relationships, the average relationship duration was 32.42 months (*SD* = 34.05).

### Measures

**MMEA-TR Short Form.** We assessed psychological IPV perpetration using the short form of the MMEA-TR. For a detailed description of the instrument, please refer to Study 1.

**The Lesbian Internalized Homophobia Scale.** To assess lesbians and bisexual individuals’ internalized heterosexism, we used the Attitudes Toward Other Lesbians subscale of the Lesbian Internalized Homophobia Scale, originally developed by Szymanski and Chung (2002) and later adapted into Turkish by Öztürk and Kındap (2011). To make the subscale inclusive for bisexual participants, we modified the items of the subscale by adding the term “bisexuals” (e.g., “I have respect and admiration for other lesbians/bisexuals”). The original subscale consists of 8 items, rated on a 7-point Likert scale ranging from 1 (*strongly disagree*) to 7 (*strongly agree*), with higher scores indicating greater internalized heterosexism. However, in the Turkish adaptation, two additional items (i.e., “Social situations with other lesbians make me feel uncomfortable” from the Connection With the Lesbian Community subscale and “Lesbian couples should be allowed to adopt children the same as heterosexual couples” from the Moral and Religious Attitudes Toward Lesbians subscale) also loaded onto this subscale, resulting in a 10-item version. Szymanski and Chung (2002) reported the Cronbach’s alpha coefficient of .77 for this subscale, while Öztürk and Kındap (2011) demonstrated the Cronbach’s alpha coefficient of .78.

### Procedure

We employed a procedure comparable to that used in Study 1, with two primary distinctions: ethical approval was granted by the Institutional Ethical Review Board of MEF University, and participants in this study were not provided access to free counseling services.

Following ethical approval from MEF University (the Institutional Ethical Review Board), data were collected during the spring and summer of 2019 using a purposive sampling procedure via an online survey. Although ethical approval was obtained from a Danish institution, the Turkish version of the survey adhered to the same ethical standards, which were explicitly stated in the informed consent form. Participation was voluntary, and all participants provided informed consent. The survey included a notice alerting participants that some items might elicit emotional responses and encouraging them to consider their mental well-being before proceeding.

Participants were recruited through LGBTQ+ organizations, queer and feminist groups, and NGOs in Turkey. Most organizations shared the survey link on their social media accounts, and some sent it directly to their members, with reminders sent once per month over four months. This extensive outreach was intended to ensure representative samples from the target populations. The survey took approximately 10–15 min to complete, and participants were thanked and provided with study information upon completion. The response rate could not be estimated because Google Forms only provides access to completed responses, without information on how many individuals received but did not complete the survey. Participants were selected based on four inclusion criteria: (1) voluntary participation, (2) age over 18, (3) gender identity defined as a woman and sexual orientation defined as lesbian or bisexual, and (4) having a current or previous romantic relationship (dating, cohabiting, engaged, or married).

### Data Analysis

The statistical methods and tools applied in Study 3 mirrored those used in Study 1. For further details, please see the data analysis section outlined in Study 1.

## Results

### Confirmatory Factor Analysis

We found an overall mediocre yet acceptable model fit:* χ*^*2*^(98, *N* = 178) = 273.00, *p* < .001, *χ*^*2*^*/df* = 2.78, CFI = .854, TLI = .822, RMSEA = .092 (90% CI = .086-.114), and SRMR = .068. Although the CFI and TLI values were below the commonly accepted cutoffs, modification indices did not indicate any notable model misfit or underperforming items. As presented in Table 5, standardized factor loadings ranged from .481 to .695 for Restrictive Engulfment, .661 to .719 for Denigration, .471 to .845 for Hostile Withdrawal, and .649 to .823 for Dominance/Intimidation, all statistically significant at *p* < .001. These results confirm that each item reliably reflected its corresponding latent factor.

**Table 5:** Factor loadings of confirmatory factor analysis (Study 3; *N* = 178)

Factors	Indicator	Estimate	SE	Lower	Upper	Std Estimate
Restrictive engulfment	RE_1	.461	.065	.333	.590	.543
	RE_2	.825	.087	.653	.998	.695
	RE_3	.701	.115	.476	.927	.481
	RE_4	.499	.057	.386	.613	.646
Denigration	DEN_1	.962	.101	.763	1.162	.661
	DEN_2	.502	.050	.402	.602	.694
	DEN_3	.607	.059	.489	.724	.719
	DEN_4	.520	.049	.422	.617	.717
Hostile withdrawal	HW_1	1.479	.126	1.132	1.727	.798
	HW_2	1.599	.127	1.349	1.849	.845
	HW_3	.618	.101	.418	.817	.471
	HW_4	1.064	.131	.807	1.322	.598
Dominance/Intimidation	DOM_1	1.134	.092	.952	1.316	.823
	DOM_2	.471	.052	.368	.573	.649
	DOM_3	.856	.079	.701	1.011	.753
	DOM_4	.688	.073	.544	.832	.669

Lower and upper refer to unstandardized estimates that are within the 95% confidence intervals

### Correlation Analysis

As displayed in Table 6, all sub-factors of the MMEA-TR short form showed significant and positive intercorrelations. The strongest correlation was observed between Denigration and Dominance/Intimidation (*r* = .74), whereas the weakest was between Restrictive Engulfment and Hostile Withdrawal (*r* = .44). Furthermore, internalized heterosexism demonstrated significant positive associations with each subscale of the MMEA-TR short form, with correlation coefficients ranging from *r* = .24, *p* < .01 for Hostile Withdrawal to *r* = .41, *p* < .01 for Restrictive Engulfment.

**Table 6:** Correlation matrix (Study 3; *N* = 178)

Variables	1	2	3	4	5	6
1. Restrictive engulfment	–					
2. Denigration	.60**	–				
3. Hostile withdrawal	.44**	.48**	–			
4. Dominance/Intimidation	.56**	.74**	.44**	-		
5. Psychological violence	.76**	.83**	.80**	.81**	–	
6. Internalized heterosexism	.41**	.40**	.24**	.29**	.40**	–
Cronbach alpha	.68	.79	.77	.81	.89	.67

Psychological violence was computed as the sum of the four subscales (Restrictive Engulfment, Denigration, Hostile Withdrawal, and Dominance/Intimidation) to represent overall psychological IPV perpetration

^*^ *p* < .05, ** *p* < .01, *** *p* < .001

### Reliability Analysis

The Cronbach’s alpha values for the MMEA-TR short form subscales were .686 for Restrictive Engulfment, .79 for Denigration, .77 for Hostile Withdrawal, and .81 for Dominance/Intimidation.

## Discussion

Study 3 provided additional support for the construct and concurrent validity of the MMEA-TR short form among lesbian and bisexual women. The expected four-factor structure was retained, and subscales demonstrated generally acceptable reliability, with only a slight reduction in one subscale’s internal consistency. The significant correlations among psychological IPV dimensions again indicate that they represent related yet distinct forms of psychological IPV. Notably, the positive association between internalized heterosexism and psychological IPV perpetration aligns with minority stress theory, suggesting that internalized stigma may heighten emotional strain and contribute to psychologically violent behaviors within lesbian and bisexual women’s intimate relationships.

### Study 4

#### Purpose of Study and Hypotheses

In Study 4, our purpose was to establish initial evidence for the construct validity, concurrent validity, and internal consistency reliability of the MMEA-TR short form among a sample of men. We formulated hypotheses for concurrent validity.

##### ***Hypothesis 7***

All potential sub-factors of the MMEA-TR short form will be correlated with one another among a sample of predominantly heterosexual men.

##### ***Hypothesis 8***

Fragile masculinity will be correlated with all the potential sub-factors of the MMEA-TR short form among a sample of predominantly heterosexual men.

## Method

### Participants

The study sample comprised 160 men aged 18–30 (*M* = 24.49, *SD* = 2.535). Regarding sexual orientation, 92.5% identified as heterosexual, 5% as gay, 1.2% as bisexual, and 1.3% chose not to disclose. Employment status was as follows: 52.8% were employed, 20.5% were unemployed and students, 15.5% were both employed and students, 9.9% were unemployed, and 1.3% selected “other”. In terms of education, 72.7% had a university degree, 24.2% were high school graduates currently pursuing university education, and 3.1% had only completed high school. The relationship status distribution was as follows: 39.1% were not currently in a romantic relationship but had prior experience, 34.2% were currently dating, 10.6% were engaged, 10.6% reported a more complicated status, and 5.5% were married. Among participants in a relationship, the mean relationship duration was 16.09 months (*SD* = 24.324).

### Measures

**MMEA-TR Short Form.** To assess psychological IPV perpetration, the short form of the MMEA-TR was utilized. Detailed information about the measure is reported in Study 1.

**Perceived Threat to Manhood Scale.** To gauge the fragile masculinity of emerging adult men, the Threat to Subordination to Women subscale of the Perceived Threat to Manhood Scale, developed by Türkoğlu (2013) to measure university students’ perceived threat to masculinity, was employed. This subscale consists of 15 items (e.g., “Having a wife/partner who is educated better than you”). Responses are rated on a 7-point Likert scale, ranging from 1 (very uncomfortable) to 7 (extremely comfortable), with lower scores indicating a greater perceived threat to masculinity. Türkoğlu (2013) reported a Cronbach’s alpha coefficient of .92 for this subscale.

### Procedure

Ethical approval for this study was obtained from the MEF University Ethics Committee. Data were collected between October and December 2023 using an online survey administered via Google Forms. A convenience sampling method was employed. The survey link was distributed through the social media accounts (e.g., Instagram, Twitter) of the researchers. Participants were provided with an informed consent form outlining the study’s purpose, procedures, inclusion criteria (e.g., being a man aged 18–30 with prior romantic relationship experience), potential risks and benefits, the option to withdraw at any time without penalty, and the requirement for voluntary participation. They were also informed that participation would take approximately 10–15 min. Participation was voluntary and anonymous. Data were accessible only to the researchers to ensure confidentiality.

### Data Analysis

In Study 4, we employed the same statistical procedures as those used in Study 1. Details regarding the data analysis are provided in the corresponding section of Study 1.

## Results

### Confirmatory Factor Analysis

The model yielded evidence of an overall moderate yet acceptable fit to the data, χ^2^(98, *N* = 160) = 359.00, *p* < .001, χ^2^/df = 3.66, CFI = .801, TLI = .756, RMSEA = .097 (90% CI = .090–.115), and SRMR = .074. Although the CFI and TLI values did not reach the commonly recommended benchmarks, the examination of modification indices did not reveal any notable areas of model misfit or problematic items. As presented in Table 7, all standardized factor loadings were statistically significant at *p* < .001, ranging from .451 to .640 for Restrictive Engulfment, .738 to .789 for Denigration, .443 to .883 for Hostile Withdrawal, and .564 to .804 for Dominance/Intimidation. Collectively, these results provide support for the adequacy of the model, indicating that each observed indicator reliably represented its corresponding latent construct.

**Table 7:** Factor loadings of confirmatory factor analysis (Study 4; *N* = 160)

Factors	Indicator	Estimate	SE	Lower	Upper	Std Estimate
Restrictive engulfment	RE_1	.76	.10	.56	.97	.64
	RE_2	.92	.10	.66	1.17	.62
	RE_3	.83	.10	.56	1.11	.51
	RE_4	.42	.08	.26	.58	.45
Denigration	DEN_1	.97	091	.79	1.14	.74
	DEN_2	.65	.05	.54	.77	.78
	DEN_3	.68	.06	.55	.81	.73
	DEN_4	.60	.05	.50	.71	.78
Hostile withdrawal	HW_1	1.58	.11	1.35	1.81	.88
	HW_2	1.57	.12	1.33	1.81	.85
	HW_3	.63	.11	.41	.86	.44
	HW_4	1.36	.13	1.09	1.63	.71
Dominance/Intimidation	DOM_1	1.09	.10	.89	1.28	.77
	DOM_2	.56	.05	.46	.67	.78
	DOM_3	.79	.06	.65	.93	.80
	DOM_4	.66	.09	.49	.84	.56

Lower and upper refer to unstandardized estimates that are within the 95% confidence intervals

### Correlation Analysis

As shown in Table 8, all subscales of the MMEA-TR short form demonstrated significant positive intercorrelations. Fragile masculinity was also positively and significantly associated with each subscale of the MMEA-TR short form, with correlation coefficients ranging from *r* = .11, *p* < .05 for Hostile Withdrawal to* r* = .30, *p* < .001 for Denigration.

**Table 8:** Correlation matrix (Study 4; *N* = 160)

Variables	1	2	3	4	5	6
1. Restrictive engulfment	–					
2. Denigration	.47***	–				
3. Hostile withdrawal	.55***	.52***	–			
4. Dominance/Intimidation	.37***	.67***	.49***	–		
5. Psychological violence	.75***	.79***	.86***	.76***	–	
6. Fragile masculinity	.22**	.30***	.11*	.20**	.27**	–
Cronbach alpha	.64	.85	.82	.83	.89	.84

Psychological Violence = Psychological violence was computed as the sum of the four subscales (Restrictive Engulfment, Denigration, Hostile Withdrawal, and Dominance/Intimidation) to represent overall psychological IPV perpetration

^*^ *p* < .05, ** *p* < .01, *** *p* < .001

### Reliability Analysis

The internal consistency coefficients (Cronbach’s alpha) for the MMEA-TR short form subscales were calculated as .64 for Restrictive Engulfment, .85 for Denigration, .82 for Hostile Withdrawal, and .83 for Dominance/Intimidation.

## Discussion

Findings from Study 4 further support the factorial and concurrent validity, as well as the reliability of the MMEA-TR short form in a sample of men. The scale retained its original four-factor structure, with acceptable item loadings and subscale intercorrelations indicating related yet distinct dimensions of psychological IPV. Internal consistency coefficients were generally adequate across subscales, with Restrictive Engulfment showing slightly lower reliability. Additionally, fragile masculinity was positively associated with psychological IPV perpetration subdimensions, consistent with theoretical accounts suggesting that psychological IPV perpetration in men may be partly driven by stress related to maintaining culturally prescribed masculine roles.

## General Discussion

We aimed to assess the preliminary construct validity, concurrent validity, and internal consistency reliability of the short form of the MMEA-TR across four independent samples consisting of bisexual individuals (Study 1), predominantly heterosexual women (Study 2), lesbian and bisexual women (Study 3), and predominantly heterosexual men (Study 4) in Türkiye.

### Construct Validity

Across four distinct samples (bisexual individuals, predominantly heterosexual women, lesbian and bisexual women, and predominantly heterosexual men), CFA supported the factorial validity of the short form of the MMEA-TR. In all samples, the model yielded mediocre but acceptable fit indices, and all items significantly loaded onto their intended latent factors, with nearly all factor loadings exceeding the .30 threshold. Only in Study 1, item 2 (“Threatened to hit the other person”) under Dominance/Intimidation had a loading below .30. However, the item still demonstrated significant loading (*p* < .05) on its respective factor, and its removal did not improve model fit, suggesting its theoretical and empirical relevance. These findings indicate that the short form of the MMEA-TR retains the original factorial structure (Maldonado et al., 2022; Murphy & Hoover, 1999) and functions consistently across diverse gender and sexual orientation groups. Importantly, this consistency across samples is noteworthy given the sociocultural variability in the expression and recognition of psychological IPV. In collectivist, patriarchal, and heteronormative societies such as Türkiye (Uskul & Cross, 2019), the perception and normalization of psychologically abusive behaviors may differ substantially across subgroups, particularly among sexual minorities who often navigate additional layers of marginalization (minority stress; Meyer, 2003). The fact that the scale demonstrated structural validity across these varied groups highlights its cross-cultural applicability and supports its utility for assessing psychological IPV perpetration within diverse relational and identity contexts.

Across four independent samples, and consistent with our hypotheses (H1, H3, H5, H7), all sub-factors of the MMEA-TR short form were significantly correlated, providing support for the concurrent validity of the scale. This pattern indicates that, although conceptually distinct, the subdimensions (i.e., Restrictive Engulfment, Denigration, Hostile Withdrawal, and Dominance/Intimidation) capture interconnected facets of psychological IPV experiences among bisexual, predominantly heterosexual women, and lesbian and bisexual women, as well as predominantly heterosexual men in Türkiye. These associations are in line with theoretical perspectives that highlight the intertwined nature of psychological abuse tactics within intimate relationships (Maldonado et al., 2022; Murphy & Hoover, 1999; Toplu-Demirtaş et al., 2018). Collectively, these findings offer preliminary evidence that the MMEA-TR short form operates as a coherent, multidimensional tool for assessing psychological IPV across diverse sexual and gender identities.

### Reliability

Internal consistency reliability analyses across four distinct and diverse samples generally indicated that the MMEA-TR short-form subscales yielded reliability coefficients above the commonly accepted threshold of .70 (Nunnally, 1978). While a few instances (Study 1, Dominance/Intimidation = .63; Study 2, Restrictive Engulfment = .65; Study 3, Restrictive Engulfment = .68; Study 4, Restrictive Engulfment = .64) produced alpha values in the .60–.70 range, these coefficients are still considered acceptable, particularly within the context of social and behavioral sciences, where constructs are often complex and multifaceted (Aiken, 2000).

### Concurrent Validity

Consistent with our second hypothesis, jealousy was significantly linked to all sub-factors of the short form of the MMEA-TR in the bisexual sample. This finding resonates with both international and Turkish literature, which portrays jealousy as a potent relational cognition closely connected to psychologically abusive behaviors in intimate relationships (Keilholtz et al., 2023; Zurnacı & Toplu-Demirtaş, 2025). Specifically, the association between jealousy and Restrictive Engulfment suggests that bisexual individuals experiencing higher jealousy levels may be more likely to attempt to monitor, restrict, or control their partner’s social environment. This supports previous research indicating that jealousy often motivates behaviors aimed at reducing perceived threats to monosexism, especially in non-heteronormative or marginalized relationships where insecurity may be heightened due to societal stigmatization (e.g., Zurnacı & Toplu-Demirtaş, 2025). The associations between jealousy and Denigration and Hostile Withdrawal further indicate that jealousy may also fuel emotionally distancing or belittling tactics, possibly as attempts to express unmet attachment needs (e.g., Velotti et al., 2018, 2020). This is particularly relevant for bisexual individuals, whose relationships are frequently subjected to negative stereotypes such as being unstable, hypersexual, or untrustworthy (McCole & Anderson, 2025; Zurnacı & Toplu-Demirtaş, 2025), which can intensify relationship conflicts and emotional reactivity. Lastly, the significant link with Dominance/Intimidation suggests that jealousy can also manifest in more overtly threatening or coercive behaviors, indicating a broader range of psychological IPV associated with jealousy.

In line with our fourth hypothesis, we found positive correlations between anxious attachment and all sub-factors of the MMEA-TR short form among predominantly heterosexual women, which mirrors the literature linking anxious attachment to psychological IPV perpetration (e.g., Murphy & Hoover, 1999; Toplu-Demirtaş et al., 2018). Individuals with higher levels of anxious attachment tend to be more sensitive to rejection, experience intense fear of abandonment, and hyperactivate their attachment needs, which may lead to psychologically abusive behaviors within romantic relationships (Velotti et al., 2018, 2020). These tendencies seem to manifest as various forms of psychological IPV, including Restrictive Engulfment, Denigration, Hostile withdrawal, and Dominance/Intimidation.

Consistent with our sixth hypothesis, internalized heterosexism was positively associated with psychological IPV perpetration among lesbian and bisexual women. This finding supports prior research (e.g., Badenes-Ribera et al., 2019; Balsam & Szymanski, 2005; Ummak et al., 2022), suggesting that negative internalized attitudes toward one’s sexual orientation can contribute to psychological aggression. From the minority stress perspective (Meyer, 2003), internalized heterosexism seems to function as a proximal stressor, increasing psychological vulnerability and interpersonal tension. Lesbian and bisexual women with higher levels of internalized heterosexism may experience greater distress or self-criticism, which can manifest as psychological aggression toward their partners.

Finally, as predicted in our eighth hypothesis, fragile masculinity demonstrated significant positive associations with several subdimensions of psychological IPV perpetration in Study 4, a pattern consistent with previous research highlighting the role of masculine insecurity in aggressive behaviors within intimate relationships (e.g., Reidy et al., 2014; Türkoğlu, 2013). Specifically, fragile masculinity was linked to attempts to restrict and control a partner’s autonomy (Restrictive Engulfment), to demean or belittle the partner (Denigration), and to exert coercion and intimidation (Dominance/Intimidation). Although its association with Hostile Withdrawal was comparatively weaker, this finding nonetheless suggests that fragile masculinity may also be expressed through more passive forms of relational disengagement and emotional avoidance. From the lens of Gender Role Stress Theory (O’Neil, 1981), the associations between fragile masculinity and psychological IPV perpetration subdimensions highlight how masculine insecurity may operate as a “psychological vulnerability”. Men who experience distress when they feel they are not meeting cultural standards of toughness, control, or authority may channel this insecurity into attempts to reassert dominance over their partners (Eisler & Blalock, 1991; Moore et al., 2010). For instance, controlling behaviors (Restrictive Engulfment) and belittling (Denigration) can be seen as compensatory strategies for coping with the internal discomfort of failing to meet masculine expectations. Similarly, intimidation and coercion (Dominance/Intimidation) may serve as behavioral attempts to mask or defend against feelings of inadequacy. Even the weaker link with Hostile Withdrawal may reflect an avoidant response to gender role stress, choosing disengagement as a way to escape the threat of being unmasked as insufficiently masculine. Precarious Manhood Theory (Vandello & Bosson, 2013) may also help explain these findings as expressions of men’s “reactive sensitivity” to threats against their manhood. If manhood is a precarious and socially tenuous status that must be continually defended, then fragile masculinity may reflect heightened reactivity (such as intimate partner violence, Reidy et al., 2014) to such challenges. In this framework, controlling, belittling, and intimidating behaviors appear to function not only as coping mechanisms but also as performative defense*s*, visible demonstrations of masculinity aimed at re-establishing social legitimacy. Hostile Withdrawal, though weaker, may also fit here, as withdrawal represents a way to avoid situations that could expose vulnerability, thereby protecting the masculine image.

### Limitations and Further Suggestions

This study should be interpreted in light of several limitations. First, the relatively small sample sizes in the four studies may limit the generalizability of the findings, underscoring the need for larger, more diverse participant groups in future research to yield more generalizable results. In addition to this, although the four studies were conducted at different time points and for different purposes, we cannot fully rule out the (very unlikely) possibility that some participants may have participated in more than one study. Second, the use of convenience sampling via online surveys may have excluded individuals with limited internet access or lower digital engagement. To improve representativeness, subsequent studies should complement online recruitment with offline strategies, such as outreach through community centers, universities, healthcare facilities, and local NGOs. Third, reliance on self-report data may have introduced recall errors and social desirability bias, particularly regarding IPV perpetration. In addition to the general limitations of self-report data, the present study may be affected by common method variance, which can inflate observed associations between variables, and by acquiescent response style, where some participants may tend to agree with items regardless of their content. Incorporating partner reports or other informant-based measures could mitigate these biases. Fourth, focusing solely on perpetration precluded examination of victimization, underscoring the importance of assessing both perpetration and victimization in future studies. Fifth, measurement invariance could not be evaluated due to the independence and relatively small size of the subsamples; recruiting larger, stratified samples would allow for invariance testing across gender identities. Sixth, predictive validity and temporal stability were not assessed, suggesting the value of longitudinal research incorporating test–retest reliability analyses. Seventh, the cross-sectional design limits causal inference; longitudinal approaches could be used to clarify relationships and changes over time. Finally, convergent and discriminant validity with a broader range of external measures was not investigated. Future research should address this by conducting comparative validity analyses with other psychological IPV instruments and theoretically related constructs to further strengthen the evidence base for the MMEA short form.

### Practical Implications for Mental Health Professionals

The validated MMEA-TR short form offers mental health professionals a brief and inclusive tool for identifying psychological IPV, including relational patterns that may be subtle, normalized, or minimized in clinical settings. Its four-factor structure (Restrictive Engulfment, Denigration, Hostile Withdrawal, and Dominance/Intimidation) supports a more precise assessment of psychological IPV across diverse sexual orientations and gender identities.

The MMEA-TR can be incorporated into intake or early sessions, particularly when clients present relational- and identity-based distress. Findings across samples indicate that psychological IPV is linked to different psychosocial vulnerabilities (e.g., jealousy, attachment insecurity, internalized heterosexism, and fragile masculinity), suggesting that case formulations should be adapted to clients’ relational histories and identity contexts rather than relying on a uniform model.

Mental health professionals can use specific subscale patterns to inform the focus of their interventions. For example, the Restrictive Engulfment may signal a fear of abandonment and difficulty tolerating autonomy, Denigration may reflect attempts to manage self-worth through criticism, Hostile Withdrawal may indicate emotional over-regulation or conflict avoidance, and Dominance/Intimidation may emerge when emotional vulnerability feels threatening. Interventions may include enhancing emotional awareness, refining communication skills, increasing tolerance for relational uncertainty, and developing alternative coping strategies for managing distress.

Given the influence of heteronormativity, gender role expectations, and stigma in Türkiye, mental health professionals should consider how cultural norms shape harmful relational behaviors. The MMEA-TR provides language for discussing psychological IPV in a clinically precise and culturally responsive way, supporting non-pathologizing dialog with diverse clients.

### Conclusion

In conclusion, this study provides the first Turkish validation of the short form of the MMEA and represents the first non-heteronormative validation of a psychological measure of IPV, demonstrating its applicability across both heterosexual and LGB individuals. Using multiple independent samples, the study provides robust preliminary evidence for the reliability and validity of the MMEA short form across diverse populations. By focusing specifically on psychological IPV perpetration, it also contributes to a relatively underexplored area of the literature and introduces a practical, psychometrically sound tool to support future research and intervention efforts across various relational contexts.

## Data Availability

Data are not publicly available but can be obtained upon a reasonable request.

## Code Availability

Code availability (software application or custom code) is not applicable.
